# Stromal Derived Factor-1 (SDF-1) and Its Receptors CXCR4 and CXCR7 in Endometrial Cancer Patients

**DOI:** 10.1371/journal.pone.0084629

**Published:** 2014-01-09

**Authors:** Malgorzata Walentowicz-Sadlecka, Pawel Sadlecki, Magdalena Bodnar, Andrzej Marszalek, Pawel Walentowicz, Alina Sokup, Arnika Wilińska-Jankowska, Marek Grabiec

**Affiliations:** 1 Department of Obstetrics and Gynecology, The Ludwik Rydygier Collegium Medicum in Bydgoszcz, the Nicolaus Copernicus University of Torun, Bydgoszcz, Poland; 2 Department of Clinical Pathology, The Ludwik Rydygier Collegium Medicum in Bydgoszcz, the Nicolaus Copernicus University of Torun, Bydgoszcz, Poland; 3 Department of Gastroenterology, Angiology and Internal Diseases, The Ludwik Rydygier Collegium Medicum in Bydgoszcz, the Nicolaus Copernicus University of Torun, Bydgoszcz, Poland; 4 Department of Physiotherapy, The Ludwik Rydygier Hospital in Torun, Torun, Poland; University of Nebraska Medical Center, United States of America

## Abstract

**Purpose:**

One of the most important function of stromal derived factor-1 (SDF-1) and its receptors, is regulating the process of metastasis formation. The aim of our study was to investigate the correlation between SDF-1, CXCR4 and CXCR7 protein levels measured by immunohistochemistry with the clinicopathological features and the survival of endometrial cancer patients.

**Materials and Methods:**

92 patients aged 37–84 (mean 65.1±9.5) were enrolled to our study between January 2000 and December 2007. After the diagnosis of endometrial cancer, all women underwent total abdominal hysterectomy, with bilateral salpingoophorectomy and pelvic lymph node dissection. In all patients clinical stage (according to FIGO classification), histologic grade, myometrial invasion, lymph node and distant metastases were determined.Furthermore, the survival time was assessed. Immunohistochemical analyses of SDF-1, CXCR4 and CXCR7 were performed on archive formalin fixed paraffin embedded tissue sections.

**Results:**

Statistically significant correlations (p<0.01) were reported between SDF-1 and the clinical stage of disease, lymph node metastases, distant metastases, deep myometrial invasion (≥50%), cervical involvement, involvement of adnexa. Statistically significant correlation (p<0.01) was found between SDF-1 expression and the risk of the recurrence. Higher SDF-1 expression was associated with ahigher risk of recurrence (p = 0.0001). The results of CXCR4and CXCR7 expression didn't reveal any significant differences(p>0.05) between the proteins expression in the primary tumor cells and the clinicopathological features. Moreover, the Kaplan-Meier analyses demonstrated a stepwise impairment of cancer overall survival (OS) with increasing SDF-1 expression.

**Conclusion:**

The important role of SDF-1 as a predictor of negative clinicopathological characteristics of atumor suggests that the expression of this stromal factor should be included in the panel of accessory pathomorphological tests and could be helpful in establishing a more accurate prognosis in endometrial cancer patients.

## Introduction

Conditions in which tumor cells are able to initiate local or distant spreadare very important but still unknown clinical issue. Tumor growth and ability to initiate metastases are connected with the continuous interaction between cancer cells and the host's microenvironment. The tumor microenvironment plays an important role in each step of cancer development. There is continuous interaction between cancer cells and extracellular matrix (ECM), with fibroblasts, endothelial cells and inflammatory cells [1]. Several studies have indicated that the tumor microenvironment responds to factors derived from the tumor cells, as well as vice versa and in consequence has an impact on the neoplastic transformation[2]. The microenvironment could be involved in promoting the mutagenesis of tumor cells as well as in modification of the tumor stroma compartment. Neoplastictransformationleads to phenotypicmodifications in stromalcells[3],[4].

Endometrialcancer (EC) is the sixth most commonlydiagnosedcanceramongwomenglobally, with approximately 288,000 newcases and 50,327 deathsoccurringworldwideeachyear.However,among all the cancers of the female reproductive system, endometrial cancer became the most common with women in Europe and the United States[5]. 5125 new ECcases were diagnosed in 2010 in Poland, what makes endometrial cancer the third most common cancer, after breast and lung cancer in the Polish population [Polish National CancerRegister].

The natural course of endometrial cancer is slow and the disease is characterized by a rather good prognosis. An early onset of clinical symptoms enables to set the diagnosis at the early stage of the disease. The 5-year overall survival (OS) rate of women with endometrial cancer is more than 80% for all stages and more than 90% for stage I[6]. Endometrial cancer is successfully treated with surgery and/or radiotherapy [7]. However, for patients with an advanced or recurrent disease, or for those who wish to preserve their fertility, treatment options arelimited. However, there is a group of patients with a poor prognosis, who will benefit from more aggressive treatment. This group will need adjuvant chemo- or radiotherapy. The research of the predictive factors of recurrence or death is of great importance.

Recognized poor prognostic factors for endometrial cancer are advanced FIGO stage, a non-endometrioid histological subtype, high grade (G3), deep invasion of myometrium (>50%), presence of lymph node metastasis, cervical involvement and lymphovascular space invasion (LVSI)[6]. All risk factors mentioned above are identified after extensive surgical procedure.

Even though our knowledge of tumor cells has improved a lot throughout recent years, the precise mechanisms that control the process of metastases formation remain unknown. Chemokines belong to a superfamily of small (7–16 kDa), proinflammatory cytokines, which were originally characterized by their properties of inducing migration of leukocytes [8]. They were proven to play an important role in many biological processes connected with inflammation, such as embryogenesis, angiogenesis, atherosclerosis and cancer. It is known that the molecular level of cancer is caused by impaired inflammatory response.Tumor growth and its ability to initiate metastases are connected with the continuous interaction between cancer cells and the host's stroma [4].

Stromal derived factor -1 (SDF-1), also known as CXCL 12 plays a crucial role in inflammation and hematopoesis by acting as a chemoattractant of cells involved in inflammation and stem cells migration[9].SDF-1 encoding gene is located on the chromosome 10.q.11.1 [10].SDF-1 has two major isoforms, α and β. Both are derived from a single gene, due to alternative splicing. SDF-1 α is the predominant isoform, secreted by bone marrow stromal cells and endothelial cells and is found in nearly all organs[10]. The chemokine SDF-1 is an important α-chemokine that binds primarily to its cognate receptor CXCR4 and thus regulate the trafficking of normal and malignant cells [11]. It was believed that CXCR4 was the only one receptor for SDF-1, but recent studies have demonstrated that SDF-1 also binds to another receptor called CXCR7[12], [13]. CXCR4 and CXCR7 mediate tumor metastasis in several types of cancer (e.g. breast, lung cancer, lymphoma) [14].

One of the most important functionsof SDF-1 and its receptors, is regulating the formation of metastasis. Chemokine receptors may potentially facilitate tumor dissemination at each of the crucial steps of metastasis, including the adherence of tumor cells to endothelium, extravasation from blood vessels, metastatic colonization, angiogenesis, proliferation, and the escape from the host's response through the activation of certain pathways, such as ERK/MAPK, PI-3K/AKT/mTOR, or JAK/STAT and others. The growing evidence exists, that chemokines, including SDF-1, facilitate communication between cancer cells and non-neoplastic cells in the tumor microenvironment, promoting the infiltration, and the activation of tumor-associates macrophages and neutrophils in the stroma [15].

Because the SDF-1/CXCR4/CXCR7 axis plays a critical role in cancer development, progress, metastasis and recurrence, we hypothesize that SDF-1 and its receptors CXCR4 and CXCR7 might also be connected with the outcome of treatment of endometrial cancerpatients. Any factor that gives us more knowledge about the tumor's aggressiveness would be crucial in determining the extent of the treatment.It would be of special interest to know the specific molecular mechanisms responsible for the development of lymph node and distant metastases, and local spread of the tumor. When we have become more familiar with the molecular mechanisms underlying this process, we may be able to underline the necessity of target regimens, that can modify or inhibit these processes. The purpose of our study was to investigate the correlation between SDF-1, CXCR4 and CXCR7 protein levels measured by immunohistochemistry with theclinicopathological features and survival of endometrial cancer patients.

## Materials and Methods

The study was approved by the authors' institutional review board.The Ethical Committee at the LudwikRydygier Collegium Medicum, Nicolaus Copernicus University of Torun approved this study protocol (decision No KB 332/2007). All participants have provided theinformed, written consent. The patients' baseline characteristics were anonymously taken to document the process. Ethics committees approve this consent procedure.

### Patients

Ninety two patients with endometrial cancer, aged 37–84 (mean 65.1±9.5) were enrolled to our study between January 2000 and December 2007. After the diagnosis of endometrial cancer based on specimens obtained from curettage, all patients underwent total abdominal hysterectomy, with bilateral salpingoophorectomy and pelvic lymph node dissection performed by experienced gynecological oncologists at the Department of Oncologic Gynecology of LudwikRydygier Collegium Medicum in Bydgoszcz, Nicolaus Copernicus University.

The clinical stage was assessed based on the evaluation of the surgical specimens performed by two independent experienced pathologists according to the International Federation of Gynecology and Obstetrics (FIGO) 2009 system.

The study group included 27 patients with stage IA, 18 with stage IB, 14 with II, 10 women with stage IIIA, 17 with IIIC and 6 with IV. Histological grade was assessed according to the WHO classification. Histological grade 1 (G1) was noted in 7 patients, G2 in 66 and G3 in 19 women. Deep myometrial invasion (>50%) was observed in 36 patients, lymph node metastases in 23 women, distant metastases in 6, cervical involvement in 38 and adnexal involvement in 11 patients. Baseline characteristics of the study group are enclosed in Table 1.

**Table 1:** Characteristics of endometrial cancer patients.

		N (%)
FIGO stage	IA	27 (29.35%)
	IB	18 (19.57%)
	II	14 (15.22%)
	IIIA	10 (10.87%)
	IIIC	17 (18.48%)
	IVB	6 (6.52%)
Grading	G1	7 (7.61%)
	G2	66 (71.74%)
	G3	19 (20.65%)
Bokhman subtype	Endometrioid(I)	70 (76.09%)
	Non-endometrioid (II)	22 (23.91%)
Lymphnode metastases (N)	Absent N0	69 (75%)
	Present N1	23 (25%)
Distant metastases (M)	Absent M0	86 (93.48%)
	Present M1	6 (6.52%)
Myometrial invasion	<50%	56 (60.87%)
	≥50%	36 (39.13%)
Cervical Involvement	Absent	54 (58.70%)
	Present	38 (41.30%)
Infiltration of adnexa	Absent	81 (88.04%)
	Present	11 (11.96%)

All patients were divided into three risk factor groups: low risk – FIGO IA, G1 or G2, Bokhman type I (endometrioid); intermediate risk – IA G3, IB G1 or G2, Bokhman type I (endometrioid); high risk – all patients in type II (non-endometrioid), IB G3, FIGO II and higher. Patients from the low risk group did not receive any further treatment after surgery, women from the intermediate risk group received brachytherapy (VBT) 5 weeks after surgery, and patients from the high risk group underwent teleradiotherapy and VBT. Adjuvant chemotherapy was administered to ten patients with non-endometrioid histopathological subtype (chemotherapy consisted of carboplatin and paclitaxel).

In all cases, overall survival was determined (in months). Among all the cases only patient proven death related to cancer were analyzed. The follow-up time was 60–80 months.

### Methods

The studies were performed on archive formalin fixed paraffin embedded tissue sections derived from the Department of Clinical Pathomorphology Collegium Medicum Nicolaus Copernicus University.

To establish immunohistochemical procedures, a series of positive control reactions were performed. The positive controls were performed on a model tissue, for whichthe presence of the analyzed antigens was indicated in reference sources (The Human Protein Atlas), as well as in manufactured antibodies datasheet, (Table 2). The negative control reactions were performed on additional studies as well as on control tissue sections during proper immunohistochemical staining, with substituting the primary antibody by the solution of diluted 1% BSA (bovine serum albumine) in PBS (phosphate buffered saline).

**Table 2:** Antibody characteristic.

Antibody	Firm catalog number	Primary antibody dilution	Positive control according to Human Protein Atlas	Cellular localization/Expression
ANTI-SDF-1 alpha Rabbit polyclonal	AB9797	1∶200	PlacentaTrophoblastic cells	Secreted Nuclear/cytoplasmic
ANTI-CXCR4 Rabbit polyclonal	AB7199	1∶500	PlacentaTrophoblastic cells/decidual cells	Cell membraneCytoplasmic/membranous
ANTI-CXCR7Rabbit polyclonal	AB38089	1∶100	KidneyCells in tubules	Cell membraneCytoplasmic/membranous

The immunohistochemical studies were performed according to standard protocol using rabbit polyclonalantibodies against: SDF-1 (ab9797, Abcam, Cambridge, UK), CXCR4 (ab7199, Abcam), CXCR7 (ab38089, Abcam). The staining of SDF-1α and CXCR7 were performed automatically in Dako AurostainerLink48, and CXCR4 staining was performed manually.Epitopes were unmasked using Epitope Retrieval Solution pH-9 in PT-Link (Dako, Glostrup, Denmark). The endogenous peroxidase activity was blocked using Peroxidase Block (Dako) for 10 minutes, and the non-specific antibody binding was blocked by 5% BSA (bovine albumin solution) in PBS (Phosphate Buffered Saline). Tissue sections were incubated with primary antibody against SDF-1 and CXCR7 for 30 min. in RT (room temperature) and overnight at 4°C with anti-CXCR4. An antigen-antibody complex was detected using EnVisionFLEX-HRP (Dako), and localizedaccording tothe presence ofa brownreaction productusing DAB (3-3′diaminobenzidine) as achromogen.

#### Evaluation of protein expression

The studies were done at 20x original objective magnification for each of the studied antibodiesusing alight microscopeECLIPSEE800(Nikon Instruments Europe, Amsterdam, Netherlands).

The protein immunohistochemical expression was estimated using morphometric principles based on theRemmele-Stegner scale[16][IRS: 0–12], according to ratio of the intensity of protein expression (scale (0–3); 0 – negative, 1 – low staining, 2 – moderate staining, 3 - strong staining) and the positively expressed number of cells or tissue area (scale (0-4); 0, negative; 1, <10% positive area; 2, 10–50% positive area; 3, 50–80% positive area; 4, ≥80% positive area).

The pathologists who were evaluating the immunohistochemical expression of examined antigens worked independently, and they have been blinded for the patients' clinical as well as other data.

#### Statistical analysis

All statistical analysis were performed using thePQStat version 1.4.4.126.The statistical significance of SDF-1, CXCR4 and CXCR7 correlation in relation to clinicopathologicalfeatures was assessed using theKruskal-Wallis and the U Mann-Whitney test. P value <0.05 was considered statistically significant.

The overall survival rate was examined for significance using the log-rank test and the Kaplan-Meier curves. Moreover, univariate and multivariate Cox regressions were performed.

## Results

**Figure 1:**
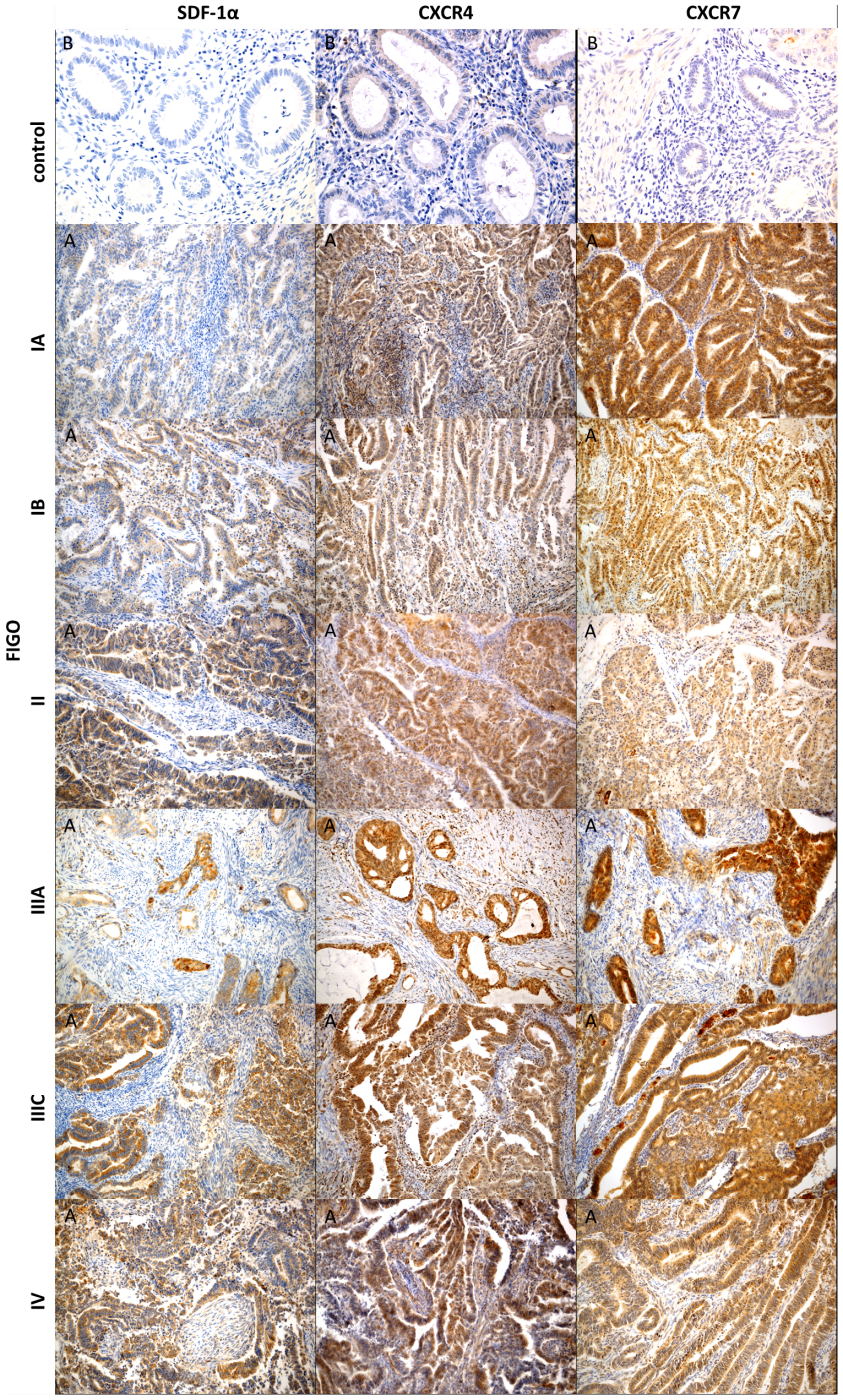
Immunohistochemical representative microphotographs representing the SDF-1, CXCR4 and CXCR7 expression in endometrial cancer according to FIGO classification (IA, IB, II, IIIA, IIIC, IV). (A) Primary objective magnification 10×.(B) Primary objective magnification 20×.

We have evaluated the expression of SDF-1, CXCR4 and CXCR7 in the primary tumor cells.The expression of SDF-1 was found in 90% cases, the expression ofCXCR4 and CXCR7was found in 100%of all cases of endometrial cancer (Figure 1).Analysis of expression evaluated in adjacent normal tissue revealed very low expression (IRS  = 1) of CXCR4 in normal endometrium. Furthermore, there was no expression of SDF-1 and CXCR7 in selected areas of normal endometrium (IRS  = 0) (Figure1).

The results obtained for each factor according to the patients' histopathological features are shown in table 3–5.

**Table 3:** SDF-1 in relation to clinicopathological features.

		Mean	Standard deviation	Minimum	Lower quartile	Mediana	Upper quartile	Maximum	p	
FIGO stage	IA	2.69	1.64	0	2	3	4	6	a	<0.0001
	IB	4.35	2.15	0	4	4	6	8	ab	
	II	4.00	2.35	0	2	4	6	8	ab	
	IIIA	5.00	2.71	2	4	4	6	12	ab	
	IIIC	5.06	1.20	3	4	6	6	6	b	
	IVB	7.33	2.42	6	6	6	8	12	b	
Grading	G1	3.14	1.57	0	2	4	4	4		Ns
	G2	4.34	2.50	0	3	4	6	12		
	G3	4.22	1.63	2	3	4	6	8		
Bokhman subtype	Endometrioid (I)	3.93	2.00	0	3	4	6	8		Ns
	Non-endometrioid (II)	5.14	2.90	0	4	6	6	12		
Lymphnod metastases	Absent (N0)	3.76	2.26	0	2	4	6	12		0.0005
	Present (N1)	5.57	1.88	3	4	6	6	12		
Distant metastases	Absent (M0)	4.00	2.13	0	3	4	6	12		0.0013
	Present (M1)	7.33	2.42	6	6	6	8	12		
Myometrial invasion	<50%	3.55	2.32	0	2	4	6	12		0.0002
	≥50%	5.29	1.84	2	4	6	6	12		
Cervical infiltration	Absent	3.60	1.96	0	2	4	4	8		0.0025
	Present	5.08	2.46	0	4	6	6	12		
Infiltration of adnexa	Absent	3.90	2.00	0	3	4	6	8		0.0046
	Present	6.55	2.98	4	4	6	8	12		
Risk of recurrence	Low	2.60	1.70	0	2	2.5	4	6		0.0001
	Intermediate and high	4.69	2.24	0	4	4	6	12		

**Table 4:** CXCR4 in relation to clinicopathological features.

		Mean	Standard deviation	Minimum	Lower quartile	Median	Upper quartile	Maximum	p
FIGO stage	IA	5.64	1.68	2	4	6	6	9	Ns
	IB	5.59	1.80	2	4	6	6	9	
	II	5.21	1.85	2	4	6	6	9	
	IIIA	5.20	1.40	2	4	6	6	6	
	IIIC	5.53	2.15	2	4	6	6	9	
	IVB	6.17	2.48	3	4	6	9	9	
Grading	G1	6.14	1.46	4	6	6	6	9	Ns
	G2	5.52	1.80	2	4	6	6	9	
	G3	5.33	2.06	2	4	5	6	9	
Bokhman subtype	Endometrioid (I)	5.42	1.71	2	4	6	6	9	Ns
	Non-endometrioid (II)	5.86	2.14	2	4	6	8	9	
Lymphnod metastases	Absent (N0)	5.47	1.68	2	4	6	6	9	Ns
	Present (N1)	5.70	2.20	2	4	6	8	9	
Distant metastases	Absent (M0)	5.48	1.78	2	4	6	6	9	Ns
	Present (M1)	6.17	2.48	3	4	6	9	9	
Myometrial invasion	<50%	5.44	1.69	2	4	6	6	9	Ns
	≥50%	5.66	2.03	2	4	6	6	9	
Cervical infiltration	Absent	5.76	1.75	2	4	6	6	9	Ns
	Present	5.21	1.89	2	4	6	6	9	
Infiltration of adnexa	Absent	5.50	1.79	2	4	6	6	9	Ns
	Present	5.73	2.15	2	4	6	6	9	
Risk of recurrence	Low	5.42	1.46	2	4	6	6	9	Ns
	Intermediate and high	5.56	1.92	2	4	6	6	9	

**Table 5:** CXCR7 in relation to clinicopathological features.

		Mean	Standard deviation	Minimum	Lower quartile	Median	Upper quartile	Maximum	p
FIGO stage	IA	6.74	2.71	2	6	6	8	12	ns
	IB	5.82	1.33	2	6	6	6	9	
	II	5.43	2.68	2	4	6	6	12	
	IIIA	7.90	2.73	4	6	7.5	9	12	
	IIIC	6.88	2.32	3	6	6	9	12	
	IVB	4.67	1.51	3	3	5	6	6	
Grading	G1	5.57	2.15	2	4	6	6	9	ns
	G2	6.43	2.27	2	6	6	6	12	
	G3	6.53	3.17	2	4	6	9	12	
Bokhman subtype	Endometrioid (I)	6.33	2.51	2	6	6	6	12	ns
	Non-endometrioid (II)	6.55	2.32	3	6	6	6	12	
Lymphnod metastases	Absent (N0)	6.38	2.53	2	6	6	6	12	ns
	Present (N1)	6.39	2.27	3	6	6	9	12	
Distant metastases	Absent (M0)	6.51	2.47	2	6	6	8	12	ns
	Present (M1)	4.67	1.51	3	3	5	6	6	
Myometrial invasion	<50%	6.18	2.46	2	6	6	6	12	ns
	≥50%	6.71	2.46	3	6	6	9	12	
Cervical infiltration	Absent	6.45	2.37	2	6	6	6	12	ns
	Present	6.29	2.61	2	4	6	9	12	
Infiltration of adnexa	Absent	6.39	2.39	2	6	6	7	12	ns
	Present	6.36	3.04	3	4	6	6	12	
Risk of recurrence	Low	7.00	2.70	2	6	6	8.5	12	ns
	Intermediate and high	6.21	2.38	2	6	6	6	12	

Statistically significant correlation (p<0.01) wasreported between SDF-1expresion and the clinical stage of the disease. Higher expression was observed throughout increasing FIGO stages. However, there were no statistically significant correlations between histological grade (G) and Bokhman subtype of endometrial cancer (Table 3).

According to the lymph node involvement higher SDF-1 expression was obtained in the primary tumor of patients with lymph node metastasis compared to patients without lymph node metastasis (p = 0.0005). SDF-1 expression was significantly higher in patients with lymph node metastases (N1), distant metastases (M1), deep myometrial invasion (≥50%), cervical involvement,and involvement of adnexa (Table 3).

Statistically significant correlation (p<0.01) was found between SDF-1 expression and the risk of the recurrence. Higher SDF-1 expression was associated with the higher risk of recurrence (p = 0.0001) (Table3).

The results of CXCR4and CXCR7 expressionsdidn't reveal any significant correlation (p>0.05) between the proteinsexpression in the primary tumor cells and theFIGOclinical stage, grading, as well as the Bokhmansubtype, and lymph nodes and distant metastases (Table 4, 5).Even so, statistical analyses didn't reveal any significantcorrelationbetween CXCR4 and CXCR7expressions and deep myometrial invasion (≥50%), cervical and/or adnexalinfiltration,andthe risk of recurrence (Table 4, 5).

**Figure 2:**
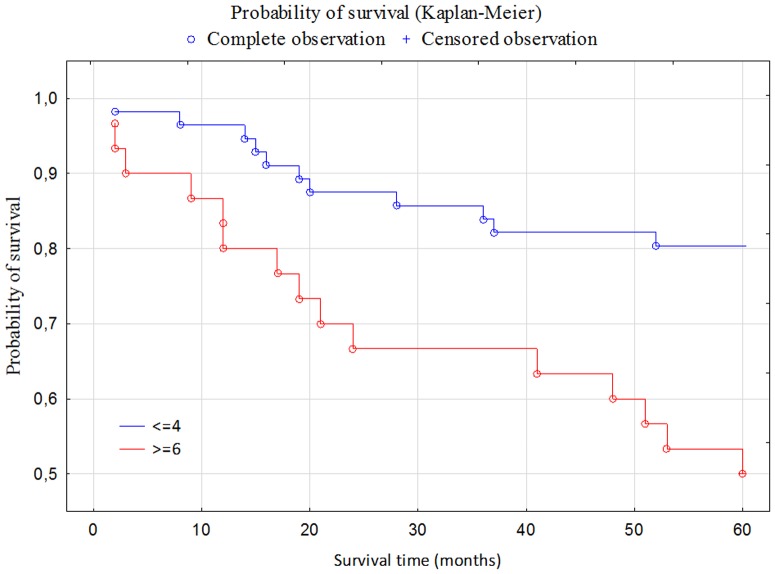
Kaplan-Meier curves of endometrial cancer patients survival in relation to SDF-1 expression.

Moreover, the Kaplan-Meier analyses demonstrated a stepwise impairment of cancer overall survival (OS) with increasing SDF-1 expression. The Log-rank test showed statistically significant correlation between SDF-1 expression and survival;in the group of patients with the high expression of SDF-1shorter survival was reported(p = 0.00099) (Figure 2).

The associationbetweenSDF-1 expressionand survivalrate was alsoperformed according to theunivariable and multivariable Cox regression analysis (including variables such as: advanced FIGO stage, high grade (G2+G3), non-endometrioid subtype (Bokhman II), lymph node metastases and deep myometrial infiltration (≥50%)(Table 6).

**Table 6:** Prognostic factors for overall survival selected by Cox's univariate analysis.

	Parameter evaluation	p – value	HR (hazard ratio)	HR (95%Cl)	
				−95% CI	+95% CI
SDF-1	0.2039	0.0032	1.2262	1.0708	1.4040
Figo (III +IV)	−0.395332	0.024740	0.453543	0.227453	0.904367
Grade(G2 +G3)	−7.58063	0.989416	0.540437	0.254360	1.148265
Bokhman (II)	−0.077849	0.709808	0.855817	0.376899	1.943288
Lymph node metastases (N+)	−0.413810	0.021431	0.437088	0.215933	0.884750
Myometrial invasion ≥50%	−0.546877	0.002647	0.334957	0.164164	0.683439

SDF-1 expression, advanced FIGO stage, lymph node infiltration and deep myometrial invasion were proven statistically important prognostic factors in univariate analysis for overall survival. Grade and non-endometrioid subtype were not important in the univariate analysis (Table 6). In multivariate analysis only SDF-1 expression and deep myometrial invasion was proven conditionally important (Table 7).

**Table 7:** Prognostic factors for overall survival selected by Cox's multivariate analysis.

	Parameter evaluation	p – value	HR (hazard ratio)	HR (95%Cl)	
				−95% CI	+95% CI
SDF-1a	0.0919	0.067698	1.182757	0.987860	1.416107
Figo (III +IV)	0.3167	0.582153	1.417021	0.409425	4.904305
Grade(G2 +G3)	633.7089	0.990670	0.59494	0.199397	1.78
Bokhman (II)	0.2197	0.796079	1.120251	0.473472	2.650551
Lymph node metastases (N+)	0.2926	0.485267	0.664731	0.211125	2.092921
Myometrial invasion ≥50%	0.2326	0.086169	0.450121	0.180869	1.120198

## Discussion

Neoplasm is composed of cells in which alterations are reflected by impaired growth control mechanisms. The cancer cells exist in a specific microenvironment, which exerts a significant effect on the various stages of tumor development. There are numerous interactions, not only between the cancer cells but also between the extracellular matrix (ECM) and fibroblasts, endothelial, and inflammatory cells.

It should be remembered that neoplasm represents a systemic condition, which is associated with disorders involving numerous components of the tumor and its stroma, which interactions constitute a prerequisite for the process of invasion and formation of metastases [17].

The results of many studies confirmed the important role of the stromal factor SDF-1 in tumor development. Increased expression of SDF-1 in fibroblasts, and especially the expression of CXCR4 receptor on the membranes of cancer cells, particularly in the hypoxemic areas of the tumor, imply the growth, mobility, and invasiveness of the malignancy. Cancer associated fibroblasts (CAF) were proven to stimulate the progression of malignancy through the release of SDF-1. In turn, SDF-1 causes the recruitment of progenitor endothelial cells, stimulating angiogenesis. Tumor metastases into areas rich in SDF-1 are associated with the expression of CXCR4 on the surface of cancer cells and their uptake to SDF-1-rich sites along a concentration gradient. This is reflected by the invasion of cancer cells to the site of metastasis and resultant destruction of surrounding tissues. Additionally, the expression of CXCR4 is modulated by the stroma, which is also associated with progression of the tumor [17].

The expression of SDF-1 signaling pathways, modulated by its specific receptors, especially CXCR4 was well established[18]. The activation of CXCR4occurs in multiple signalling pathways, and consequentlye leads to variety biological cell responses [19]. It was clearly proven that CXCR4 is the target for negative regulation connected withG-protein signaling which is able to regulate the CXCR4 expression in several ways. Two pathways were identified. First the G-protein dependent signalling linked to transcription and expression through the PI3K-AKT-NF-κβ, MEK1/2 and Erk1/2 signal transduction. Secondly the G-protein independent signalling pathway via JAK/STAT investigated.Numerous studies indicate the role of receptor CXCR7 in SDF-1 activation, as the G-protein independent pathway [20]. But theresults of aforementioned reports partlystill remains unclear. Lee et al.evaluatedthe migration of melanocytes toward SDF-1. The authors investigated that SDF-1 induces the migration of melanocytes through CXCR7, and not CXCR4. The authors also established that the SDF-1/CXCR7 complex promotes the activation of the β-arrestin 2-dependent signaling pathway. Therefore, it is very important to search for appropriate SDF-1signalling pathways through CXCR (both CXCR4 and CXCR7) in different cancers that would suggestnew therapeutic strategies.

The mechanisms by means of which SDF-1/CXCR4/CXCR7 pathway enhances the progression of malignancy remain unexplained. A number of studies involving tumor models, especially ovarian cancer, were conducted to answerthis question. It was confirmed that SDF-1 stimulates angiogenesis, mostly through enhancing the synthesis of VEGF. Perhaps, SDF-1 promotes the survival of cells through the activation of signaling cascades of PI3 and MAP kinases, without the cell cycle progression. SDF-1 modulates apoptosis via Bcl-2/BAD, and MEK, S6, and PI3K kinase pathways. Furthermore, genes whichexpressions are associated with enhanced survival of cells can be activated through exposure to SDF-1. Therefore, SDF-1 can promote the survival of cells by means of two mechanisms: post-translational inactivation of apoptosis, and enhanced transcription of genes associated with cell survival. More aggressive phenotype of SDF-1-positive tumors can be associated with increased synthesis of metalloproteinases and enhanced transcription of genes responsible for the activation of metabolic pathways promoting the invasiveness and formation of metastases[21], [22], [23].

The results of sparse published studies dealing with the role of SDF-1/CXCR4/CXCR7 pathway in endometrial cancer are contradictory and inconclusive. The aim of this study was to analyze the expression of SDF-1, CXCR4, and CXCR7 proteins in primary endometrial cancer.

In this study, the expression of SDF-1 was documented in 90% of the patients, while all of them showed the presence of CXCR4 and CXCR7 receptors. We have proven that the expression of SDF-1 increased proportionally to the FIGO stage (p<0.001). In contrast, we did not observe statistically significant correlationsbetweenSDF-1 expression and the degree of histological differentiation, or various types of tumor according to Bokhman's classification. Patients with lymph node involvement, distant metastases, infiltration of the cervical stroma, and infiltration of myometrium exceeding 50% of its thickness showed significantly higher levels of SDF-1 expression. The expression of SDF-1 proved a significant predictor of the presence of these negative prognostic factors in endometrial cancer patients.

In contrast, no significant associations were documented between the expression of CXCR4 and CXCR7 proteins and the clinicopathological characteristics of endometrial cancer; similarly, these parameters were not proven to play the role of negative prognostic factors of this malignancy.

The number of published reports on the prognostic value of the components of SDF-1/CXCR4/CXCR7 pathway in endometrial cancer patients is sparse. Furthermore, the results of these studies are inconclusive and sometimes contradictory.

Mizokami et al.[24]analyzed the relationshipbetween the degree of histologicaldifferentiation, animportantprognosticfactor of endometrialcancer, and the expression of SDF-1 protein and CXCR4 in 41 patientsaffected with thismalignancy. The authors revealed an inverse correlation between the degree of histological differentiation and the expression of both SDF-1 and CXCR4. The stromal expressions of SDF-1 and CXCR4 did not prove to be associated with the tumor grade.

Kodama et al. claimed that there is the association between CXCR4 expression and the clinical stage of the tumor [25]. The authors analyzed the group of 55 endometrial cancer patients and revealed that higher clinical stages of this malignancy are associated with lower levels of CXCR4 expression. They observed decreased expression of CXCR4 in patients with higher FIGO stages, deep myometrial invasion, lymph node and adnexal metastases, and more aggressive phenotype of the tumor. Furthermore, similar to our study, the authors revealed that CXCR4 does not constitute an independent predictor of survival.

The results of these two studies were contradictive to findings documented in patients with other malignancies (breast, prostate and lung cancer), and did not confirm the association of SDF-1/CXCR4 signaling pathway with a more aggressive phenotype of tumor, as the expression of proteins involved in this pathway proved lower in tumors characterized by worse prognosis [15].

Another study of the role of SDF-1/CXCR4 signaling pathway was conducted by Tsukamoto et al.[26]. These authors analyzed the relationship between SDF-1 and CXCR4 expression and the tumor's potential for myometrial invasion. The depth of myometrial infiltration represents an important prognostic factor in endometrial cancer patients, as the deep invasion is more often associated with the presence of distant metastases and lymph node involvement. The authors examined five lines of endometrial cancer cells and material obtained from 34 patients with this malignancy [24]. The average level of CXCR4 expression was higher in patients in whom the infiltration exceeded 50% of myometrial thickness than in women without deep myometrial invasion. Experiments with endometrial cancer lines revealed that SDF-1 activates PIK3/Akt signaling pathway, which is necessary for the processes of endometrial cancer cell migration. This study was the first to suggest that SDF-1α/CXCR4 axis plays a role in the invasion of endometrial cancer.

Another study, conducted by Gelmini et al., analyzed the presence of mRNA and the expression of SDF-1, CXCR4, and CXCR7 proteins in 41 patients operated on due to endometrial cancer[27]. CXCR7, discovered recently by Burns et al., promotes the growth and adhesion of many cancer lines in vitro[13]. Gelmini et al. determined the levels of mRNA by means of RT-PCR, and analyzed the expression of proteins using a 3-item immunohistochemical scale. They observed that the tumor level of CXCR4 mRNA was higher and the level of SDF-1 expression lower than in normal tissue. Furthermore, the CXCR4-positive cancer cells were able to migrate towards higher levels of SDF-1, which was associated with greater invasiveness of the tumor and formation of distant metastases. The authors revealed that the expression of CXCR4 in highly-differentiated tumors (G1) was decreased compared to poorly-differentiated ones (G3). In contrast, no significant correlationsassociated with the degree of histological differentiation were documented in the case of CXCR7 mRNA levels. Similar to our study, the authors did not reveal any statistically significant correlationsin the expressions of CXCR4 and CXCR7 proteins associated with the various histological grades of primary tumor[27].

A recently published study of the role of SDF-1/CXCR4 axis in endometrial cancer involved 199 patients, in whom the estrogen receptor status was examined apart from the SDF-1 and CXCR4 expressions[28]. The authors of this study did not find a significant relationship between the estrogen receptor status and the expression of SDF-1.

This evidence suggests that the role of SDF-1/CXCR4/CXCR7 axis in the progression of endometrial cancer still remains unexplained. The authors of the first two studies mentioned above reported an inverse relationship between the expressions of proteins involved in SDF-1/CXCR4 pathway and the degree of histological differentiation or clinical stage of the tumor. In contrast, according to the authors of the remaining studies, higher expression of SDF-1/CXCR4 axis components is associated with a worse prognosis. It should be noted, however, that none of the aforementioned studies analyzed the exact the same sets of prognostic factors. The studied parameters included tumor grade and FIGO stage, the depth of myometrial invasion, or the involvement of the cervix, adnexa, and lymph nodes [24], [25], [26], [27]. Secondly, the authors of these studies used various classification systems of protein expression. In our study, the expression of antibodies was assessed with the Remmele-Stegner scale, considering both the percentage of antibody-labeled cells and the intensity of their staining.

In our study, the increased expression of SDF-1 was documented in patients with negative prognostic factors of endometrial cancer, such as a high clinical stage, a lymph node involvement, a deep myometrial invasion,an infiltration of the cervix and adnexa, and the presence of distant metastases. SDF-1 proved the most important predictor of poor prognosis in endometrial cancer patients, and similar to other malignancies, increased expression of this molecule was associated with a more aggressive phenotype of the tumor. Furthermore, we provedstatistically significant differences in 5-year survival time. The survival of patients showing high expression of SDF-1 (> = 6) was significantly shorter than those with the low expression (< = 4).

Zhang et al. showed that the expressions of SDF-1 and CXCR4 in prostate cancer were higher than in the case of benign hyperplasia. Furthermore, increased expression of SDF-1 was associated with greater invasiveness of the tumor and the presence of distant metastases [29]. Also Kajiyama et al. reported that high expression of CXCR4 was reflected by markedly lower rates of 5-year survivals in ovarian cancer patients. Moreover, these authors conducted an in vitro experiment which confirmed that SDF-1 is involved in the development of peritoneal metastases, enhancing the adhesion of ovarian cancer cells to the peritoneum [30]. Liu et al. showed that increased expression of SDF-1/CXCR4 can play a vital role in the promotion of lymph node metastases. These authors revealed a positive correlation between the SDF-1 expression and the prevalence and severity of lymph node involvement [31]. Furthermore, an in vivo experiment involving an animal (murine) model showed that the intraperitoneal implantation of human endometrial cancer line HEC1A is reflected by the development of distant metastases to the lungs, liver and peritoneum. However, complete regression of the lung and liver metastases was documented in mice in which anti-CXCR4 antibody was administered, pointing to CXCR4 as a potential target of anticancer treatment.

The evidence of the vital role of stromal factor SDF-1 in the pathogenesis of endometrial cancer, and documented association between its expression and the negative histopathological prognostic factors of this malignancy, suggests that its determination could be useful in routine clinical practice. This refers mostly to the identification of patients who require more aggressive therapy due to the presence of negative prognostic factors and the increased risk of recurrence. Therefore, patients with increased expression of SDF-1 should be included in further large trials verifying the potential predictive role of this molecule. If the role of SDF-1 expression would beconfirmed in endometrial cancer patients it could be helpful in designing pharmaceutical trials aimed at the improvement of therapeutic outcomes in SDF-1-positive women.

The important role of SDF-1 as a predictor of negative clinicopathological characteristics of the tumor, suggests that the expression of this stromal factor should be included in the panel of accessory pathomorphological tests and could be helpful in establishing more accurate prognosis in endometrial cancer patients.
